# Everything is Infinite: Children’s Beliefs About Endless Space, Time, and Number

**DOI:** 10.1162/opmi_a_00104

**Published:** 2023-09-20

**Authors:** Jessica Sullivan, Sophie Cramer-Benjamin, Joseph Alvarez, David Barner

**Affiliations:** Skidmore College, Department of Psychology; Yale Child Study Center, Yale School of Medicine; University of California, San Diego, Department of Psychology

## Abstract

How do children form beliefs about the infinity of space, time, and number? We asked whether children held similar beliefs about infinity across domains, and whether beliefs in infinity for domains like space and time might be scaffolded upon numerical knowledge (e.g., knowledge successors within the count list). To test these questions, 112 U.S. children (aged 4;0–7;11) completed an interview regarding their beliefs about infinite space, time, and number. We also measured their knowledge of counting, and other factors that might impact performance on linguistic assessments of infinity belief (e.g., working memory, ability to respond to hypothetical questions). We found that beliefs about infinity were very high across all three domains, suggesting that infinity beliefs may arise early in development for space, time, and number. Second, we found that—across all three domains—children were more likely to believe that it is always possible to add a unit than to believe that the domain is endless. Finally, we found that understanding the rules underlying counting predicted children’s belief that it is always possible to add 1 to any number, but did not predict any of the other elements of infinity belief.

“*Who as a child did not lie in bed filled with a slowly mounting terror while sinking into the idea of a universe that goes on and on, for ever and ever?*” – Rudy Rucker (2005)

## INTRODUCTION

On its face, the human ability to reason about infinite quantities is puzzling given the finiteness of daily experiences. And yet, from pre-Socratic philosophers like Zeno of Elea to current day kindergarteners, humans have expressed many different forms of the idea that some things must go on forever. How might such thoughts arise? Hearing Buzz Lightyear say “To infinity, and beyond!” might provoke, for any thoughtful 1st grader, a sort of modern-day, Pixar-fueled Zen koan: what’s infinity in the first place, and (once they find that out), what could possibly be beyond it? Alternatively, children may infer infinity by learning about iterative rules, like learning that it is always possible to add 1 to any number. Or, perhaps they learn about infinity via specific culturally-transmitted messaging. Beliefs about infinity may emerge one domain at a time (e.g., for space before time), or all at once. In the present paper, we investigate the cognitive foundations of human reasoning about infinity by exploring how beliefs about infinity emerge across the domains of number, space, time. We ask whether these beliefs depend on domain-specific learning processes, or instead depend on a single insight that generalizes across domains. To our knowledge, the present study is the first to investigate young children’s intuitions about infinite time, and the first to ask how beliefs about infinite number, space, and time might be related.

Considerable research has investigated how children reason about number, space, and time, focusing largely on the acquisition of beliefs about these domains that derive from observational learning and empirical induction (de Hevia et al., 2014; Droit-Volet et al., 2007, 2008; Piaget, 1969; Shatz et al., 2010; Srinivasan & Carey, 2010; Tillman & Barner, 2015; Wagner et al., 2016). Less work, however, has asked how children acquire beliefs about infinite magnitudes within these domains, perhaps in part because it’s difficult to imagine what kinds of experiences might inform such learning. Experiences of moving around the world or staring into the night’s sky might suggest that the universe is very large, but don’t justify the inference that space extends indefinitely. What’s more, objects of infinite magnitude can’t be rendered as visual images, making them impossible to imagine as actualities, an intuition that informed Aristotle’s conclusion that space can’t be infinite (Lear, 1982; Linnebo & Shapiro, 2019; Sorabji, 2006).

If infinite magnitudes can’t be directly experienced or even imagined, where might beliefs of infinite number, space, and time come from? One early idea, first described by Archytas, a predecessor of Aristotle, is that evidence for the infinite arises from the human ability to iteratively imagine new increments in number, space, and time. Archytas wondered, “If I were at the edge of the world … could I stretch out my hand or a stick into the outer region or not?” If yes, then clearly the edge hasn’t in fact been reached. But if not, there must be *something* there to impede movement—something beyond the edge—leading to the same conclusion (see Furley, 1981; Sorabji, 1987). For Archytas, the infinity of number, space, and time were mutually related: the unfolding of time was characterized as a generative process “proceeding to the creation of the things in the world” and producing “passages or transitions by virtue of the projections of things that are brought forth from it” (Sorabji et al., 2010). Similarly, although Aristotle denied the possibility of actual infinities, he allowed for what he called “potential” infinities, based on the intuition that, as when counting, elements can always be added to a set iteratively and without end, even if humans can never reach an actual infinity (for discussion, see Nuñez, 2005).

Notably, although children never directly experience infinite magnitudes, they do acquire knowledge of rule-governed counting systems that might support reasoning of the type described by Archytas. The structure of counting exhibits a generative “successor function”, reflecting the structure of the natural numbers, whereby every number has a unique successor that is also a number—implying that numbers must be infinite. In base 10 counting systems, for example, an additive rule combines units 1–9 with decades 10–90, which in turn can be recursively embedded under hundreds, thousands, etc. The process of generating successors is only limited by knowledge of the names for larger bases (Cheung & Ansari, 2023). Remarkably, there is growing evidence that, before age 7, U.S. children come to believe that it’s always possible to add 1 to a number and that numbers have no end (Cheung et al., 2017; Gelman, 1980; Evans, 1983; Hartnett & Gelman, 1998). These beliefs about the infinity of number appear to be related to children’s mastery of the syntax underlying the generation of number words: Children who can productively count up from arbitrary points in their count list are significantly more likely to believe that numbers are infinite than children who have limited, non-productive, count lists (Chu et al., 2020). Thus, although young children may lack fully adult-like beliefs about infinity—even adults lack sophisticated understanding of the notion of infinity (e.g., Falk et al., 1986; Nuñez, 2005; Monaghan, 1986, 2001; Taback, 1975; Wistedt & Martinsson, 1996), some beliefs about infinity appear to emerge early in development, possibly as a result of learning about the structure of the count list.

While the data reported above suggest that children’s first intuitions about numerical infinity are related to their knowledge of counting, this leaves open whether children reason, like Archytas, that infinite number implies that space and time might also be infinite. Previous work provides data relevant to this question via studies of reasoning about finite quantities. For example, some studies have suggested that reasoning across the domains of number, space, and time is supported by a single, generalized magnitude system (Agrillo et al., 2013; Cantlon et al., 2009; Kadosh et al., 2005; Walsh, 2003; Sokolowski et al., 2015; Winter et al., 2015), though others have argued for at least partially distinct representational systems (Hamamouche & Cordes, 2020; Odic & Ariel, 2018). Similarly, some infant studies have suggested that domains like number, space, and time are supported by a common representational system early in life (de Hevia et al., 2014; Srinivasan & Carey, 2010), though others have argued for distinct systems (Droit-Volet et al., 2008; Odic & Ariel, 2018; Skagerlund & Träff, 2016), or that representations initially overlap but become gradually differentiated over time (Newcombe, 2014; Newcombe et al., 2015). In addition, children’s knowledge of numerical symbols is related to their ability to reason about time (Tillman & Barner, 2015; see also Hamamouch & Cordes, 2020), which often implicates the use of words that also describe space (Boroditsky, 2000; Casasanto & Boroditsky, 2008; Clark, 1973; Santiago et al., 2007; Tillman et al., 2022). Such observations suggest that the acquisition of language about number, space, and time could be related. Compatible with this, the trajectory for acquiring words about number and time appear similar: just like with number words, children’s understanding of time words involves first learning the relative ordering of the words (e.g., *twenty* comes before *forty*; a *minute* is less than an *hour*) and then later learning about the relative magnitudes they represent (e.g., *twenty* refers to a quantity half as large as *forty*; there are exactly sixty minutes in an hour; Sullivan & Barner, 2014; Tillman et al., 2018). Also, the language we use to talk about very large (and infinite) quantities may overlap across domains: a meeting might “go on forever” or “feel absolutely endless” [time], just as might a road [space], or the count list [number]. These observations suggest the possibility that the acquisition of abstract language for quantities may be common across the domains of number, space, and time.

Notably, some studies have laid preliminary groundwork for understanding how children reason about non-numerical notions of infinity. For example, early work by Piaget found that children appear to struggle to conceive of the infinite divisibility of spatial extents in some contexts (Piaget & Inhelder, 1956), and subsequent work by Smith et al. (2005) reported that intuitions about infinite divisibility of matter develop gradually between 3rd and 6th grade. Also, Smith et al. (2005) found that beliefs in the infinite divisibility of matter were correlated with beliefs about the infinite divisibility of number, though the precise causal nature of this correlation is unclear given the large age range tested, and the many developmental changes that also occur over the period tested in that study.

In the present study, we asked how children’s intuitions regarding infinite number, space, and time emerge in childhood, and whether they’re related developmentally. We imagined four potential causal relationships between beliefs about infinity across domains, which we test in the present study. First, is the possibility that intuitions about infinite space and time might emerge from intuitions about number—flowing from the acquisition of a generative counting system (Cheung et al., 2017; Chu et al., 2020). Alternatively, it’s possible that intuitions about space might emerge first, given its utility for reasoning about other quantities like number, time, and musical pitch (Lakoff & Johnson, 2008; Starr et al., 2020). Perhaps least likely, though still possible, is that beliefs about infinite time emerge first—perhaps emerging out of the cyclic and repeating structure of days and years, and/or from cultural myths surrounding ‘eternity’. Finally, it is possible that intuitions about infinite number, space, and time emerge independently, and are not causally related.

Building on the methods of past studies (Cheung et al., 2017; Evans, 1983), we probed whether children believe number, space, and time are bounded or infinite, and asked children questions such as, “Does number/space/time ever end, or does it go on forever?” This allowed us to directly compare children’s beliefs about infinity across all three domains. We also collected data on children’s understanding of the structure of counting to probe its relation to intuitions about infinity. Unlike previous work, we also measured children’s working memory, as well as their general ability to reason and talk about hypotheticals, in order to ensure that children’s performance on our infinity interview was attributable to their beliefs about infinity, and not to other factors. If learning is domain-specific and limited primarily by the concreteness or imageability of content, then we might expect reasoning about space or number to emerge first, followed later by reasoning about the arguably more abstract domain of time. However, if the main limit on reasoning about infinity is the notion of infinity itself—rather than the properties of a particular domain of content—then we might expect children’s beliefs about number, space, and time to be highly similar.

## METHOD

### Preregistration

All recruitment and exclusion choices were as preregistered (https://osf.io/694zg/?view_only=0e74097a732748d69b3bf28fabd5088e), unless otherwise noted.

### Participants

We recruited 121 participants from the U.S. between the ages of 4 and 7 years old; all participants were tested in-person. One participant was excluded because we discovered that they had participated in a pilot version of a similar study conducted by our lab. As pre-registered, we terminated data collection for five participants because they failed to provide answers during the Warm-Up phase. Three participants were excluded for failing to complete at least 80% of the task. Our final sample included *N* = 112 participants (*M*_age_ = 5;10, range = 4;0–7;11). Our lab does not collect race or gender data from child participants, although participants were recruited from a predominately White and middle-class region of the U.S. Northeast. Participants were tested in a psychology lab on campus at Skidmore College or on-location in preschools, daycares, and elementary schools in the Capital Region of NY state. All provided verbal assent, and their parents provided written consent, in keeping with the approved IRB protocol at Skidmore College.

### Stimuli and Procedure

#### Warm-Up.

The Infinity Interview began with a Warm-Up Phase, during which children were asked four questions: ‘What is the [fuzziest / spikiest / sweetest / loudest] thing you can think of?’ These questions familiarized participants with the question and answer format of the task, and ensured their ability to provide verbal answers. As noted above, children (*n* = 5) who failed to answer at least 2/4 of these questions did not continue the study.

#### Infinity Interview.

The interview consisted of five blocks. Three blocks included questions about infinity (one each for number, space, and time). Because our infinity interview required complex verbal processing and reasoning, we wanted to ensure that linguistic and cognitive factors weren’t limiting performance. To test this, we also included two blocks containing questions about hypothetical scenarios, also described below.

The Infinity Interview was a simplified version of that described in Cheung et al. (2017), which in turn were modeled on previous work (Evans, 1983; Gelman, 1980; Hartnett & Gelman, 1998). Participants were first asked to name the biggest number/space/time that they could think of. For example, in the number interview, we asked, “What’s the biggest number you could think about?” It didn’t matter, for our purposes, what children conceived of as the biggest number (or space, or time), but merely *that* they conceived of one, if only provisionally, for the purposes of the game. So, for this reason, children who didn’t volunteer responses were provided with prompts: “Is 2 the biggest number you can think about? … What about 1000? … What about a million?” Once the child had agreed to a biggest number—any number, volunteered or accepted from the script—the researcher then said, “Okay, you said *n* is the biggest number you can think about. Can we add 1 to that number?” The researcher then asked, “Do numbers ever end, or do numbers go on forever?” Between-subjects, we varied the order of the alternatives presented to the child: half of the children heard e.g., “Do numbers ever end, or do numbers go on forever”, while the other half heard “Do numbers go on forever, or do numbers end?”

Questions about spatial and temporal infinity were asked in the same format. Children named the biggest space/time they could think of, and children who failed to volunteer answers were given prompts. Specifically, for space they were asked, “Is this room the biggest space you can think about? … what about this city? … what about all the way from here to the moon?” For time, they were asked, “Is this snap [researcher snaps fingers] the biggest time you can think about? … What about a day? … What about from when the Earth was first made until now?” They then were asked if the biggest time or space they could think of could be increased by one minute/inch, and if time/space end, or go on forever.

In addition to questions about number, space, and time, there were two blocks of questions assessing children’s ability to respond to linguistically-presented hypothetical scenarios. For one question, participants saw a red block with a corner painted white. Participants were asked, “Could I add more red paint to this block?” and, “If nobody touched this block for a long time, would it stay red forever, or would it stop being red?” For the second question, participants were told that the researcher found a caterpillar outside. Participants were then asked, “Could I add more eyes to my caterpillar?” and “Will my caterpillar stay a caterpillar forever, or will it turn into a butterfly?” We designed the hypothetical scenarios to hopefully elicit both a response that it is possible to add more (i.e., more red paint could be added to the block) and that it is *not* possible to add more (i.e., it isn’t possible to add more eyes to a caterpillar), and to elicit both a response that something will stop/end (i.e., at some point a caterpillar will stop being a caterpillar) and that something will go on forever (i.e., an untouched red block will stay red). The purpose of this task was to identify whether children’s performance on the infinity interview could be explained by domain-general task demands (e.g., linguistic limitations; inability to reason about hypotheticals). Thus, participants’ responses were coded for whether they reflected comprehension. For example, for the question …would [the block] stay red forever, or would it stop being red?”, participants were credited with providing an acceptable response if they said it would stay red forever *or* if they gave plausible reasons why it wouldn’t stay red (e.g., “over time the sun will fade it and it will turn pink).

Order of blocks was counterbalanced such that there were 12 possible orders: Infinity Block 1 (⅓ of participants received space, ⅓ time, and ⅓ number), Hypothetical Block 1 (½ of participants received questions about a red block and half received questions about a caterpillar), Infinity Block 2 (again, ⅓ of participants completed each domain), Hypothetical Block 2 (½ received questions about a red block, ½ received the caterpillar questions), Infinity Block 3 (⅓ space, ⅓ time, ⅓ number). For example, a child might be presented with questions in the following order: Number-Caterpillar-Space-Red Block-Time.

### Number Measures

#### Give-a-Number.

Children were given an abbreviated version of the Give-a-Number task (Wynn, 1990, 1992), a measure of early childhood numeracy. Children were given a dozen miniature bears and asked to give the researcher *n* bears, where *n* was 6, 9, 7, or 5 (in that order). After the child gave the researcher the bears, the researcher asked, “Is that *n*? Can you count and make sure?” The researcher recorded whether the child gave the correct number of bears, and whether or not they counted correctly. Children who failed one trial but succeeded on the other three were allowed to repeat the trial they failed on. Children who succeeded on at least three of four trials were considered to be competent counters.

#### Highest Count With Prompts.

Children were asked to count as high as they could. The highest children were allowed to count was 140, at which point the game was ended. If a child made a mistake, the experimenter noted their error and provided a prompt. For example, if a child said, “14, 16”, the researcher noted the omission of 15, stopped the child, and prompted them: “Actually, the number that goes after 14 is *15*; 13, 14, 15 … What comes next?” Children who responded correctly were allowed to continue counting. Children who were unable to identify the next number, or who made two sequential mistakes were stopped, and moved onto the next task. We recorded children’s Initial Highest Count—that is, the highest they reached without making an error—and their Final Highest Count (the highest number reached, with prompts).

#### English Next Number.

This task probed children’s understanding of the successor function for familiar numbers. Children were given a number and asked to produce its successor: “In this game, I’m going to say a number, and you’re going to tell me the number that comes immediately after. So for example, if I say one, you say …?” The number “one” was used in the training trial. If the child correctly responded with the word “two”, there were 12 subsequent trials, ranging from 5–271 (1, 5, 7, 16, 24, 52, 71, 105, 107, 116, 224, 252, and 271). If the child responded that they did not know, they were asked to “take their best guess”. If a child repeated the prompt number or said a number lower than the prompt number, the researcher reminded them that they were asked to say what number comes next.

#### Novel Next Number.

This task was structured similarly to the English Next Number task, except that participants were introduced to an unfamiliar counting system, and asked to produce the next number in that unfamiliar system. This allowed us to test whether children’s performance in the Next Number task was driven purely by memorized knowledge, or might also reflect an additive rule (i.e., of adding +1–9 to the decade term). Children were told, “In this game, I am going to say a number in Mobi language, and then I want you to say what comes next in Mobi language. Ready? OK!” The prompt novel numbers were Mobi-one, Mobi-five, Mobi-seven, Mobi-two, and Mobi-nine, in that order. Performance was coded as correct or incorrect on each trial (although performance for Mobi-9, as pre-registered, was simply exploratory, since there was no obvious correct answer).

#### Corsi.

We employed an adapted version of the Corsi working memory task (Corsi, 1973; Vandierendonck et al., 2004) to measure children’s working memory. Children were shown a black board featuring squares whose tops were painted red. Next, they were told, “This is the block game. First it’s my turn. I’m going to point to some of the blocks in a certain order with my finger. As soon as I finish pointing, I’m going to say ‘your turn’, and I want you to use your finger to point to the same blocks in the same order that I did. So just watch closely, and then do what I did when it’s your turn. Ready?” The experimenter then tapped each block with their index finger for approximately 1 second each, without pauses in between blocks, at a regular rhythm. The child began by simply copying 1 block-tap; if they succeeded on at least one out of the two 1-block trials, they next were asked to copy a sequence of 2 block-taps. If they succeeded on at least one out of the two two-block sequences, they next received a 3 block sequence, etc. The game ended when the child failed both trials of a given sequence length. The experimenter allowed for self-corrections but did not ever repeat the sequence unless the child wasn’t looking. Performance was coded as correct or incorrect; children were considered to have failed a trial if they made an error at any point in the sequence.

## RESULTS

### Preliminary Analyses

#### Hypothetical Situation Interview (HSI).

We first consider the HSI, in order to ensure that general limits on children’s ability to reason about or respond to linguistically-presented hypothetical situations were not responsible for children’s performance on the infinity interview. Participants performed well on this task (*M* = 3.48/4 adult-like response), with 102/112 participants answering at least 3 of the 4 questions in an adult-like (i.e., sensible) manner. Critically, these children consistently provided answers in the format required by the infinity interview by indicating that it was possible to add more (red paint), that it was not possible to add more (eyeballs to a caterpillar), that something would continue in its current state forever (the red block would stay red) and that something would not continue forever (the caterpillar would change to a butterfly). We report infinity classification for the 10 participants who failed the HSI in the SOM; notably, most of these participants were “Full Infinity Believers” (*n* = 4–8 out of 10, depending on domain), suggesting that poor performance on the HSI task does not explain some of the weak infinity knowledge described below.

#### Give-a-Number & Highest Count.

Participants exhibited high numeracy: 98 of 112 children were classified as CP-knowers. Second, when asked to count as high as they could, children’s Initial Highest Count averaged 67 (Median = 39; Mode = 140), and 45/112 participants made no errors before reaching 100 (i.e., they mastered the count list for all one- and two-digit numbers). Mean Final Highest Count was 84 (Median = 108; Mode = 140), with 62/112 participants successfully counted to 100 after receiving prompts. While counting ability was relatively strong, our data were bimodal: 31 participants had an Initial Highest count <20, and 24 of these participants could not count higher than 20, even with feedback.

#### Order Effects.

We conducted preliminary order effects analyses, as preregistered, in order to determine whether the order of questions in our infinity interview impacted performance; we did not find reliable order effects; full reporting is available in the SOM.

#### Children’s Responses to the “Largest X” Question.

As preregistered, we coded children’s responses to “What is the largest X you can think about” for whether their responses referenced space, time, number, and/or the explicit notion of infinity. Full data are reported in SOM.

#### Infinity Interview.

Endorsement of infinity-beliefs was high for both of our infinity interview questions. Within each domain, more than three quarters of participants indicated that it was possible to always add one (Number: 81%; Space: 79%; Time: 79%). Similarly, a majority of participants indicated that each domain went on forever (Number: 60%; Space: 71%; Time: 63%). Below, we analyze these data in greater detail.

### Main Analyses

#### Planned Age Analyses.

**Figure 1.**
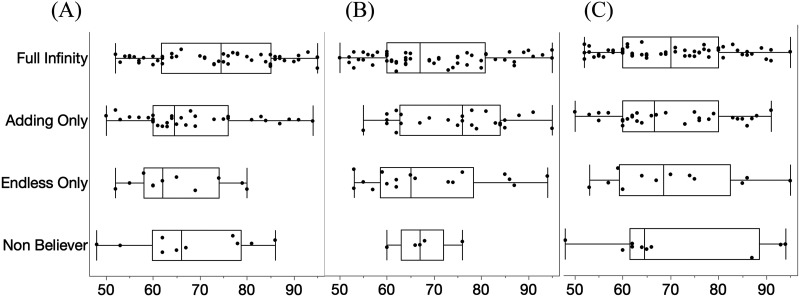
Ages of children (in months) classified into each Infinity Belief category for (A) Number, (B) Space, and (C) Time.

As preregistered, for each domain (Space, Time, Number), we asked whether there was a relationship between each component of Infinity Knowledge (belief in the ability to always add 1; belief in the domain’s endlessness) and age (continuous, in months), using logistic regression. There were no effects of age (all *p* >.10, see SOM and Figure 1 for additional visualization).

#### Infinity Interview Classification.

**Figure 2.**
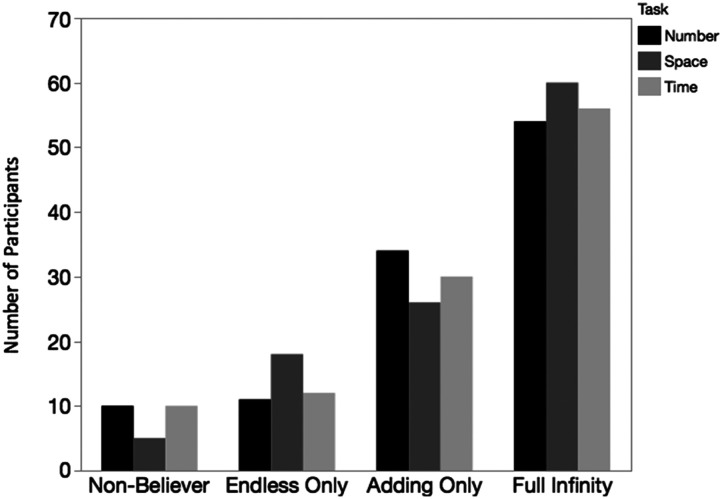
Classification by domain.

We classified children who believed that a domain had no end and that it was always possible to add a unit as Full-Infinity Believers for that domain. Full-Infinity Believers were the most frequent category for Number (49.5%). Interestingly, they were also the most frequent category for Space (55%) and Time (51.8%), resulting in nearly identical levels of Full Infinity Belief across domains (see Figure 2).

Children who believed that a domain ended but that it was always possible to add a unit were called Adding-Only Believers; this category was second most frequent for Number (31.2%), Space (23.9%), and Time (27.8%). Children who endorsed the view that a domain is endless, but did *not* believe that it is always possible to add a unit were called Endless-Only Believers, and were rare across domains (Number: 10%; Space: 16.5%; Time: 11.1%), as in previous studies on number (Cheung et al., 2017; Gelman, 1980; Hartnett & Gelman, 1998). Finally, children who believed that a domain ended *and* that it was impossible to add a unit, were called Non-Believers, and were the least frequent across all domains (Number: 9.2%; Space: 4.6%; Time: 9.3%). Participants who failed to answer enough questions within a domain to be categorized (*n* = 3–4 per domain) were classified as “Uncategorized”. Age was unrelated to infinity classification for any domain (all *p* > .05; Figure 1). In sum, we found remarkably consistent response patterns across the three domains, and found that the pattern of beliefs previously found for number are also held for space and time.

#### Consistency of Beliefs.

**Figure 3.**
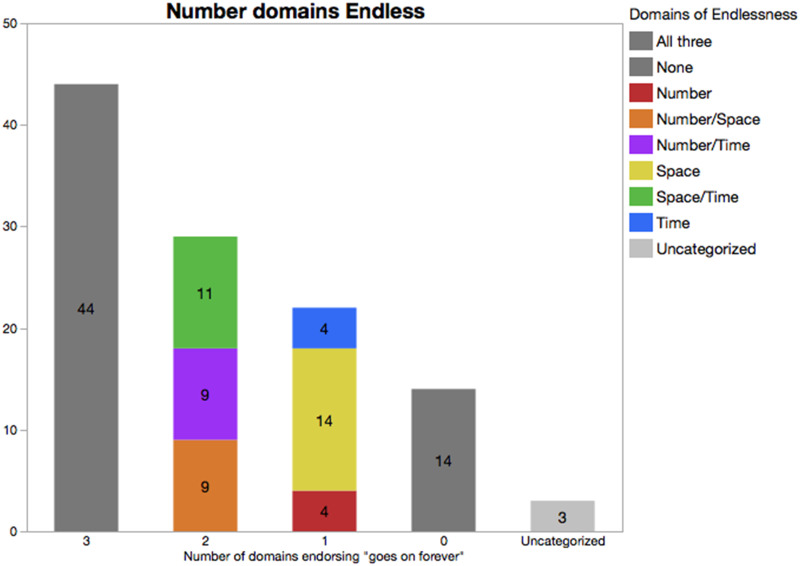
The *X*-axis indicates the number of domains for which a given child endorsed that the domain had no end. The Uncategorized label indicates that the child did not provide a response to this question for at least one domain. The *Y*-axis and embedded numerals both indicate the raw count of children; color indicates the domain.

Although the pattern described above suggests similar types of beliefs about infinity across the domains of Number, Space, and Time, these initial analyses leave open whether beliefs across domains are related *within* individual children. To explore this, we asked what percentage of children held consistent beliefs across multiple domains. For both of our measures (i.e., beliefs in endlessness and belief that it is always possible to add), the most frequent outcome was that children held consistent beliefs across all three domains. As shown in Figure 3, 65/106 (61.3%) classifiable children held consistent beliefs regarding the possibility of adding to a quantity of Number, Space, and Time. Of these, 62 believed that it was possible to add a unit for all domains, and 3 consistently said it was not possible for any domain. We also found that 58/109 classifiable children (53.2%) held consistent beliefs regarding the endlessness of domains. Of these, 44 judged that all three domains were endless, and 14 consistently judged that none of them were.

Next, we asked whether classifications (i.e., “Full Infinity”, “Endless Only”, etc.) were similar across all three domains. Out of 112 participants, 9 were unclassifiable (because they were missing data for at least one task), leaving 103 classifiable participants. Of these, 36 were classified identically all three domains, and 67 provided at least one diverging response for at least one domain. Of the 36 consistent children, 26 were “Full Infinity” believers in every domain. The remaining 10 consistent children were “Adding Only” believers across all three domains. No children were consistently Endless Only believers, and no children were consistently Non-Believers. The lack of consistent Non-Believers means that every classifiable child in our study reported at least one infinity-consistent belief. Indeed, 101/103 classifiable children provided at least 2 (out of 6 possible) infinity-consistent responses during our study.

#### Inconsistent Beliefs: Overall Patterns.

Because perfectly consistent responses make it difficult to test whether beliefs about one domain are contingent upon beliefs about another, we next consider children who performed inconsistently across domains. Of the 55 children who were Full Infinity Believers in at least 2 domains, *n* = 26 were Full Infinity Believers in the third, *n* = 14 were Adding Only Believers for the third; *n* = 13 were Endless Only Believers for the third, and *n* = 2 participants were Full Infinity Believers in two domains, and non-believers in the third. These data show that more than half of all classifiable participants provided at least 5 (out of 6) infinity-consistent responses, and therefore that most participants who were Full Infinity Believers in two domains had partial infinity belief for the third domains.

We next consider the *n* = 24 children who were Adding Only Believers across at least two domains. Of these, *n* = 10 were Adding Only Believers in the third, *n* = 10 were Full Infinity Believers for the third, *n* = 2 were Endless Only believers for the third, and *n* = 2 were Non-Believers for the third. In summary, 24 (23.3%) of the 103 classifiable participants were Adding Only Believers in at least two domains, meaning that they endorsed the belief that those domains could always be added to, but rejected the idea that those domains were endless. Only 9 children were Endless Only Believers in two domains (and, as noted below, none were Endless Only Believers in all three domains), and 6 were Non-Believers in two domains (and, again, none were Non-Believers in all three domains). In other words, it was relatively rare to find children who fit into these categories. Finally, 11 children provided truly inconsistent responses: they were classified differently in each of the three domains, and 9 were unclassifiable because they had missing data.

**Figure 4.**
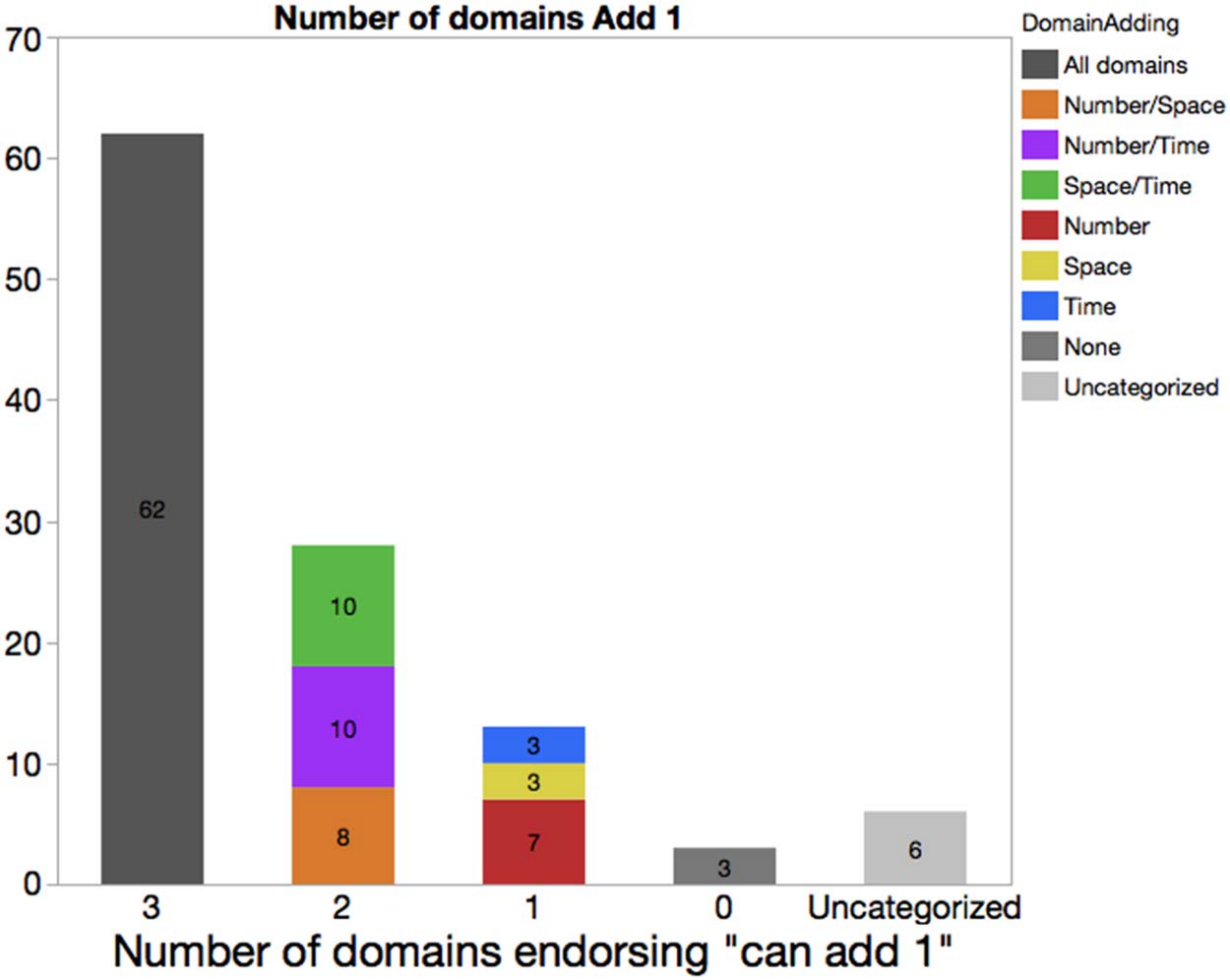
The *X*-axis indicates the number of domains for which a given child endorsed that it was possible to ‘add 1 to X’. The Uncategorized label indicates that the child did not provide a response to this question for at least one domain. The *Y*-axis and embedded numerals both indicate the raw count of children; color indicates the domain.

Next, we focused more narrowly on children’s endorsement of the belief that it is always possible to add a unit to a quantity of space, time, and/or number, and asked whether these beliefs were more likely to be held in some domains, relative to others. Of the children who believed that it was always possible to add a unit for two domains, roughly equal numbers believed that it was possible to add to just space and time but not number, or to just number and space, or to just number and time (see Figure 4). A total of 12.6% of children believed that it was possible to add one for only a single domain. While the number of children who held this belief was small (*n* = 13), more than half of these children (7/13) held this belief only for number. These data are consistent with the idea that children are more likely to hold beliefs about the possibility of always adding 1 to a number than they are to hold those beliefs for other domains. However, evidence for the primacy of number is weak in the present dataset given the small sample size.

Like in the case of adding a unit, roughly equal numbers of children believed that just space and time (but not number) were endless (*n* = 11), just number and space were endless (*n* = 9), or just number and time were endless (*n* = 9). Another 20.8% of children believed that only one domain was endless. Like in the case of adding a unit, there was an asymmetry by domain, but unlike in that case, 14/22 (64%) of those who believed that a single domain was endless held this belief for the domain of Space. Smaller numbers of children believed that only Time (*n* = 4) or Number (*n* = 4) were endless. Thus, in this case, if any domain is primary, it is space, and not number.

In sum, the relatively strong but imperfect relationship between beliefs across domains is compatible with the hypothesis that learning about infinity in one domain may impact beliefs in another, but that this may not happen in a sudden sweeping insight that affects all domains in synchrony. Also, we found that when children hold inconsistent beliefs about infinity across domains, they are somewhat more likely to hold the belief that (a) it is always possible to add 1 to the biggest number and (b) that space is endless.

#### Predictors of Beliefs About Infinity.

We next investigated predictors of children’s beliefs about infinity. If children’s beliefs were explained by learning about infinity as part of a prescribed curriculum in school, or because some cognitive capability that supports this reasoning matures at a particular age, then we might expect that age would be a strong predictor of performance. However, as noted above, age was not related to performance on any of the questions of our infinity interview (all *p* > .05; see Figure 1 and SOM). This suggests that these cross-domain relationships aren’t simply explained by the fact that older children have more adult-like beliefs.

We next asked whether performance on the Infinity Interview was related to our other measures, like numerical knowledge and working memory. To assess the relationship between beliefs in infinity and cognitive/numerical skill, we predicted each type of infinity knowledge (adding vs. endless) in separate logistic regressions from age, Initial Highest Count (as a proxy for familiarity with the count list), Final Highest Count (a proxy for the child’s generative knowledge of the count list), Novel Number performance, the English Next Number task (a measure of successor knowledge), and Corsi score.^1^ This resulted in 6 separate models (3 domains * 2 components of infinity knowledge).

We found only 1 case in which any of our predictors predicted performance on the Infinity Interview. Specifically, English Next Number performance (i.e., the number of items for which children could name that number’s immediate successor) was a significant predictor of whether participants endorsed the view that it was always possible to add 1 to the biggest number they could imagine (*B* = −.48, *SE* = .18, *p* = .007), as was age (*B* = .09, *SE* = .05, *p* = .041), when controlling for the above factors. There were no other significant predictors in any other analysis (full reporting in SOM).

## DISCUSSION

We investigated children’s beliefs about infinity in the domains of number, space, and time. We asked whether beliefs about space and time emerge along a similar timeline as beliefs about infinite number, and also whether beliefs about infinity in one domain (e.g., number), might be used to scaffold beliefs in another domain (e.g., space, time). We also measured children’s mastery of counting, on the premise that an understanding of the structure and rules of counting might lead to the intuition that numbers are infinite. Our main finding was that most children in our study believed that number, space, and time were infinite, and held generally consistent beliefs across all three domains. Also, while many children showed somewhat different levels of belief in infinity across our test items, beliefs about infinity did not systematically emerge earlier in any particular domain, leaving open whether knowledge of one domain might drive learning about another. Finally, although beliefs about infinity were related across domains, these beliefs were not consistently related to children’s knowledge of counting, and did not vary substantially according to age, raising questions about the learning mechanisms that support the development of these beliefs.

The main finding of this study was that children expressed very high levels of belief in infinity across all three domains. We replicated previous findings that children as young as 4 to 5 years of age have emerging beliefs about infinite number (Cheung et al., 2017; Chu et al., 2020), and extended them to show that beliefs about infinite space and time are also present at around this age. Specifically, more than half of our participants provided infinity-consistent responses for at least 5 out of 6 questions, and every child in our study provided at least one infinity-consistent response. Also, within each domain about half of our participants were classified as Full Infinity Believers, and a striking majority (60–81%, depending on the question and domain) of children expressed infinity-consistent beliefs on each of our infinity interview questions. These data support the conclusion that, beginning by as early as 4 years of age, English-speaking children in the US begin to exhibit explicit beliefs about the potential infinity of space, time, and number.

In addition to expressing high levels of belief in infinity overall, we found the novel result that children’s beliefs about infinite number, space, and time are relatively—but not perfectly—consistent across domains. For example, the proportion of children who were classified into a particular belief group (i.e., Full Infinity Believers, Endless Only Believers, Adding Only Believers, and Non-Believers) was nearly identical in each domain. Also, we found that about 60% of children believed that it was always possible to add a unit across all three domains, and just over 40% believed that number, space, and time were all endless.

In addition to replicating previous findings that Full Infinity and Adding Only believers are frequent while Endless Only believers are rare within the domain of number (Cheung et al., 2017; Chu et al., 2020; Evans, 1983), we add the novel result that the same pattern is true of children’s beliefs about space and time. We also found that more children believed it was always possible to add a unit (of number, space, or time) than believed that a domain was endless. According to previous studies on number, this finding may suggest that an understanding of iterative adding is the first step in learning about infinity: children may learn first that one can always add a unit, and only later recognize the entailment of this belief: that number, space, and/or time must therefore never end (see Brackett, 1998, for a discussion of transitional states of infinity belief).

While we found substantial consistency in children’s beliefs about infinity across domains, a sizable minority of children held *different* beliefs about the possibility of adding a unit across domains, and a modest majority of children held different beliefs about the endlessness of the three domains. These data suggest that it is possible for children to acquire partial beliefs about infinity, and to hold different beliefs about infinity across domains. At the outset, we asked whether acquiring beliefs about infinity in one domain automatically generalized to all other quantitative domains. Our data argue against this possibility, because a sizeable portion of our sample held differing beliefs about infinity for at least one domain. We see several possible explanations of these data. On the one hand, it may be the case that beliefs about infinity are acquired separately across domains, and that children who held differing beliefs about infinity did so because they had learned something about infinity for one domain (e.g., number) but had not yet learned it about another domain (e.g., time). On the other hand, children who expressed differing beliefs about infinity across domains may have an abstract understanding of infinity, but nevertheless believe that some domains are not infinite. For example, it is possible that children had arrived at an abstract understanding of infinity (e.g,. “the ability to iteratively add to a domain suggests that the domain is endless”) but nevertheless decided not to endorse a belief about infinity in some domains because of their beliefs about those particular domains (e.g., “while I can imagine the cases under which space might be infinite, I do not believe that space is infinite”). Future research should consider methods that may be able to differentiate whether the types of inconsistencies in beliefs that we report in the present study are best explained by (a) differing beliefs about infinity or (b) differing beliefs about the domains.

Critically, the trajectory of acquisition of beliefs about infinity is still uncertain based on our data. In order to demonstrate the trajectory for acquiring beliefs about infinity, it is important to demonstrate an initial state of non-belief. Unlike previous work that reports a sizeable group of Infinity Non-Believers for number (Cheung et al., 2017; Chu et al., 2020), such children were rare in our sample across all three domains, suggesting that our group had more advanced knowledge overall (indeed, there were no children who consistently provided infinity-non-believing responses). Relatedly, although the mean age of our sample was similar to that of previous studies, our sample included fewer children at the youngest ages, with only 10% under age 4;6. Given the importance of finding Infinity Non-Believers for detecting the order of the emergence of beliefs about infinity across domains, future studies should address the order of emergence of infinity beliefs across domains by focusing on even younger children, where it may be possible to find more Non-Believers.

At the outset, we hoped to identify whether one domain might have special status in the origin of children’s reasoning about infinity. Indeed, cases of less consistent judgments provide hints of the possible primacy of some domains. For example, among the 22 children who believed that only a single domain was endless, most of them held this belief for space. These data are consistent with the view that some children learn about the endlessness of space first, perhaps via explicit instruction (e.g., “did you know the universe is infinite?”), or perhaps because space more readily supports concrete visualizations like those described by Archytas (e.g., the possibility of always reaching one’s hand beyond any edge of space that one reaches). However, we also found that among the 13 children who believed it was possible to add a unit in only a single domain the majority held this belief only for number. These data are consistent with the possibility that children acquire beliefs about the possibility of unlimited addition for number before they do so for space or time. However, in both of these two cases just discussed, the number of children who believed in infinity for only one domain was small, limiting our ability to draw firm conclusions about causal interactions from this dataset. In addition, while there may be tentative evidence for primacy of some domains, there were many children who bucked this trend, demonstrating that there is no single entry point into beliefs about infinity.

Contrary to our initial hypothesis that children’s beliefs about infinity might be based on knowledge of how to generate successors when counting, we did not find a significant relationship between counting productivity and infinity belief outside the domain of number. While the belief that it is always possible to add 1 to any number was predicted by children’s ability to produce successors on the Next Number task (see also Chu et al., 2020), number knowledge performance didn’t predict the belief that numbers go on forever, or the belief that it is always possible to add a unit for space or time. However, because children in our sample performed relatively well on the counting tasks, our ability to detect correlations with infinity knowledge was somewhat limited. For example, almost ⅓ of children performed perfectly on the Next Number task, and ⅔ got most responses on the Next Number task correct (75% or better). Also, across domains, around 80% of children endorsed the belief that it was always possible to add 1. Therefore, as in the case of studying contingencies across domains, detecting correlations between counting and infinity knowledge may be more likely in even younger children, whose knowledge of the count list (and beliefs about infinity) may be more heterogeneous.

We found no effects of age on infinity beliefs in our study, even when we conducted post-hoc analyses restricting our dataset to the age ranges tested in previous studies (see SOM). One possible explanation for this is that, as noted above, our study may not have sampled enough young children to detect the transition from infinity-disbelief to belief. Another possibility, however, is that the trajectory from ‘lack of infinity belief’ to ‘full infinity belief’ is not linear, and not dependent only on chronological age. For example, it is possible that some individuals hold literally contradictory beliefs (e.g., that we can count infinitely upwards), but numbers are nevertheless finite (see Fischbein et al., 1979, for review), while others maintain consistent beliefs. In the spirit of our discussion of Aristotle in the Introduction, some individuals—whether children or adults—may believe that iterative processes can produce ever bigger quantities without necessarily believing that these processes result in actual infinities (i.e., iterative processes may not entail endless number, space, or time). Previous work has shown that even middle schoolers (Brackett, 1998) and college students (Tall, 1980) sometimes provide so-called ‘transitional’ responses that appear to simultaneously assert and deny the possibility of infinity, and preliminary work from our lab using reaction time methods suggests that adults may also hold internally contradictory beliefs about infinity (Cramer-Benjamin & Sullivan, in preparation). Finally, not mutually exclusive with the possibilities described above, age effects reported in previous studies of children may be due to the linguistic demands of open-ended infinity interviews, which we avoided by using a forced choice paradigm.

One critical challenge for researchers interested in studying how beliefs about infinity emerge is that we cannot see, touch, or otherwise make infinity concrete, meaning that all tasks are necessarily linguistic. First, this limits the minimum age at which we can test for infinity beliefs. While we suggest testing even younger children on infinity tasks in order to detect infinity disbelief, we also acknowledge that it is unlikely that three-year-olds have the language skills to complete such a task. Further, the role of language in tasks intended to measure infinity beliefs may also lead to challenges in interpreting the relationship between infinity beliefs across domains. This is because the language of infinity is analogous across domains (“this road/meeting/count list goes on forever!”), making it challenging to develop questions about infinity that don’t invoke multiple quantitative domains (e.g., “could I keep adding more minutes?” invokes a potentially numerical notion of units and adding). Future research should examine new ways to minimize (or systematically manipulate!) the linguistic demands of tasks aimed at measuring infinity beliefs.

To summarize, we explored the intuition of Archytas that beliefs about the infinity of space, time, and number might be deeply related to one another. Our most striking finding was that children had high levels of belief in infinity in each domain, and that these beliefs were often similar for number, space, and time. These data are consistent with the idea that children develop beliefs in infinity for space, time and number early in development, but leave open the possibility that asymmetries in these beliefs exist, and that knowledge in one domain may emerge earlier than in the others. Future research should focus on this question, while also exploring the individual differences in beliefs about infinity in adult participants, and how such beliefs might be impacted by cross-cultural differences in education, whether scientific, mathematical, or spiritual.

## ACKNOWLEDGMENTS

Special thanks to students in J.S.’s “Psychology of Infinity” course for helpful comments on the manuscript, and to Sally Apolinsky, Ajna Kertesz, Michelle Mei, and Rose Schneider for assistance with early phases of stimulus design, testing, and recruitment.

## DATA AVAILABILITY STATEMENT

Preregistration, data, and materials are available on our OSF page: https://osf.io/694zg/. Alvarez, J., Sullivan, J., Schneider, R. M., & Barner, D. (2023, March 22). Infinity Interview & Successors. Retrieved from osf.io/694zg.

## FUNDING STATEMENT

This work was supported by NSF#1749524 to J.S. and NSF #1749518 to D.B.

## Note

^1^ Note that we failed to preregister the addition of the Corsi task into this model by accident; omitting the Corsi task from the models does not substantively alter the results. Descriptives for each task are available in SOM.

## References

[bib1] Agrillo, C., Piffer, L., & Adriano, A. (2013). Individual differences in non-symbolic numerical abilities predict mathematical achievements but contradict ATOM. Behavioral and Brain Functions, 9(1), Article 26. 10.1186/1744-9081-9-26, 23815866 PMC3711901

[bib2] Brackett, J. (1998). Children’s conceptualizations of infinity: The association of mathematical context and middle-grade students’ responses to tasks involving infinity. Journal of Interdisciplinary Mathematics, 1(1), 1–31. 10.1080/09720502.1998.10700241

[bib70] Boroditsky, L. (2000). Metaphoric structuring: Understanding time through spatial metaphors. Cognition, 75(1), 1–28. 10.1016/s0010-0277(99)00073-6, 10815775

[bib72] Cantlon, J., Platt, M., & Brannon, E. (2009). Beyond the number domain. Trends in Cognitive Sciences, 13(2), 83–91. 10.1016/j.tics.2008.11.007, 19131268 PMC2709421

[bib71] Casasanto, D., & Boroditsky, L. (2008). Time in the mind: Using space to think about time. Cognition, 106(2), 579–593. 10.1016/j.cognition.2007.03.004, 17509553

[bib3] Cheung, P., & Ansari, D. (2023). A million is more than a thousand: Children’s acquisition of very large number words. Developmental Science, 26(1), Article e13246. 10.1111/desc.13246, 35170832

[bib4] Cheung, P., Rubenson, M., & Barner, D. (2017). To infinity and beyond: Children generalize the successor function to all possible numbers years after learning to count. Cognitive Psychology, 92, 22–36. 10.1016/j.cogpsych.2016.11.002, 27889550

[bib5] Chu, J., Cheung, P., Schneider, R. M., Sullivan, J., & Barner, D. (2020). Counting to infinity: Does learning the syntax of the count list predict knowledge that numbers are infinite? Cognitive Science, 44(8), Article e12875. 10.1111/cogs.12875, 32761666

[bib73] Clark, H. H. (1973). Space, time, semantics, and the child. In E. M. Timothy (Ed.), Cognitive development and acquisition of language (pp. 27–63). Elsevier. 10.1016/B978-0-12-505850-6.50008-6

[bib6] Corsi, P. M. (1973). Human memory and the medial temporal region of the brain. Dissertation Abstracts International, 34(2-B), 891.

[bib7] Cramer-Benjamin, S. & Sullivan, J. (in preparation). Unraveling infinity: Understanding the development of abstract concepts using adult speeded judgments.

[bib9] de Hevia, M. D., Izard, V., Coubart, A., Spelke, E. S., & Streri, A. (2014). Representations of space, time, and number in neonates. Proceedings of the National Academy of Sciences, 111(13), 4809–4813. 10.1073/pnas.1323628111, 24639511 PMC3977279

[bib10] Droit-Volet, S., Clément, A., & Fayol, M. (2008). Time, number and length: Similarities and differences in discrimination in adults and children. Quarterly Journal of Experimental Psychology, 61(12), 1827–1846. 10.1080/17470210701743643, 19031154

[bib11] Droit-Volet, S., Meck, W. H., & Penney, T. B. (2007). Sensory modality and time perception in children and adults. Behavioural Processes, 74(2), 244–250. 10.1016/j.beproc.2006.09.012, 17084041

[bib12] Evans, D. (1983). Understanding zero and infinity in the early school years. Publication No. (244984818) [Doctoral dissertation, University of Pennsylvania]. Dissertations available from ProQuest. https://repository.upenn.edu/dissertations/AAI8326285

[bib13] Falk, R., Gassner, D., Ben-Zoor, F., & Ben-Simon, K. (1986). How do children cope with the infinity of numbers? In Proceedings of the Tenth International Conference for the Psychology of Mathematics Education (pp. 13–18). University of London.

[bib75] Fischbein, E., Tirosh, D., & Hess, P. (1979). The intuition of infinity. Educational Studies in Mathematics, 10, 3–40. 10.1007/BF00311173

[bib14] Furley, D. J. (1981). The Greek theory of the infinite universe. Journal of the History of Ideas, 42(4), 571–585. 10.2307/2709119

[bib15] Gelman, R. (1980). What young children know about numbers. Educational Psychologist, 15(1), 54–68. 10.1080/00461528009529216

[bib17] Hamamouche, K., & Cordes, S. (2020). Learning about time: Knowledge of formal timing symbols is related to individual differences in temporal precision. Journal of Experimental Psychology: Learning, Memory, and Cognition, 46(1), 117–126. 10.1037/xlm0000714, 31021117

[bib18] Hartnett, P., & Gelman, R. (1998). Early understandings of numbers: Paths or barriers to the construction of new understandings? Learning and Instruction, 8(4), 341–374. 10.1016/S0959-4752(97)00026-1

[bib19] Kadosh, R. C., Henik, A., Rubinsten, O., Mohr, H., Dori, H., van de Ven, V., Zorzi, M., Hendler, T., Goebel, R., & Linden, D. E. J. (2005). Are numbers special? The comparison systems of the human brain investigated by fMRI. Neuropsychologia, 43(9), 1238–1248. 10.1016/j.neuropsychologia.2004.12.017, 15949508

[bib20] Lakoff, G., & Johnson, M. (2008). Metaphors we live by. University of Chicago Press.

[bib21] Lear, J. (1982). Aristotle’s philosophy of mathematics. The Philosophical Review, 91(2), 161–192. 10.2307/2184625

[bib22] Linnebo, ø., & Shapiro, S. (2019). Actual and potential infinity. Noûs, 53(1), 160–191. 10.1111/nous.12208

[bib23] Monaghan, J. (1986). Adolescents’ understanding of limits and infinity. Unpublished Ph.D. thesis, Warwick University.

[bib24] Monaghan, J. (2001). Young peoples’ ideas of infinity. Educational Studies in Mathematics, 48, 239–257. 10.1023/A:1016090925967

[bib77] Newcombe, N. S. (2014). The origins and development of magnitude estimation. Ecological Psychology, 26, 147–157. 10.1080/10407413.2014.875333

[bib25] Newcombe, N. S., Levine, S. C., & Mix, K. S. (2015). Thinking about quantity: The intertwined development of spatial and numerical cognition. Cognitive Science, 6(6), 491–505. 10.1002/wcs.1369, 26415916

[bib26] Nuñez, R. E. (2005). Creating mathematical infinities: Metaphor, blending, and the beauty of transfinite cardinals. Journal of Pragmatics, 37(10), 1717–1741. 10.1016/j.pragma.2004.09.013

[bib27] Odic, D., & Ariel, S. (2018). An introduction to the approximate number system. Child Development Perspectives, 12(4), 223–229. 10.1111/cdep.12288, 30534193 PMC6286047

[bib29] Piaget, J. (1969). The child’s conception of time. Routledge and Kegan Paul.

[bib28] Piaget, J., & Inhelder, B. (1956). The child’s conception of space. Routledge & Kegan Paul.

[bib30] Rucker, R. (2005). Infinity and the mind. Princeton University Press.

[bib31] Santiago, J., Lupáñez, J., Pérez, E., & Funes, M. J. (2007). Time (also) flies from left to right. Psychonomic Bulletin & Review, 14(3), 512–516. 10.3758/BF03194099, 17874598

[bib33] Shatz, M., Tare, M., Nguyen, S. P., & Young, T. (2010). Acquiring non-object terms: The case for time words. Journal of Cognitive Development, 11(1), 16–36. 10.1080/15248370903453568, 22259310 PMC3258973

[bib34] Skagerlund, K., & Träff, U. (2016). Number processing and heterogeneity of developmental dyscalculia: Subtypes with different cognitive profiles and deficits. Journal of Learning Disabilities, 49(1), 36–50. 10.1177/0022219414522707, 24598147

[bib35] Smith, C. L., Solomon, G. E. A., & Carey, S. (2005). Never getting to zero: Elementary school students’ understanding of the infinite divisibility of number and matter. Cognitive Psychology, 51(2), 101–140. 10.1016/j.cogpsych.2005.03.001, 16081058

[bib36] Sokolowski, A., Li, Y., & Wilson, V. (2015). The effects of using exploratory computerized environments in grades 1 to 8 mathematics: A meta-analysis of research. International Journal of STEM Education, 2(1), 1–17. 10.1186/s40594-015-0022-z

[bib38] Sorabji, R. (1987). Infinity and creation. In Philoponus and the rejection of Aristotelian science (pp. 164–178). Cornell University Press.

[bib37] Sorabji, R. (2006). Time, creation and the continuum: Theories in antiquity and the early middle ages. University of Chicago Press.

[bib39] Sorabji, R., Chadwick, H., Hoffman, P., Wolff, M., Zimmermann, F., Furley, D., & Schmidt, C. (2010). Philoponus and the rejection of Aristotelian science: Second edition. Bulletin of the Institute of Classical Studies, 103, iii–306. Retrieved from https://www.jstor.org/stable/44216227

[bib40] Srinivasan, M., & Carey, S. (2010). The long and the short of it: On the nature and origin of functional overlap between representations of space and time. Cognition, 116(2), 217–241. 10.1016/j.cognition.2010.05.005, 20537324 PMC2900540

[bib41] Starr, A., Cirolia, A., Tillman, K., & Srinivasan, M. (2020). Spatial metaphor facilitates word learning. Child Development, 92(3), e329–e342. 10.1111/cdev.13477, 33355926 PMC8169516

[bib42] Sullivan, J., & Barner, D. (2014). Inference and association in children’s early numerical estimation. Child Development, 85(4), 1740–1755. 10.1111/cdev.12211, 24397891

[bib43] Taback, S. (1975). The child’s concept of limit. In M. F. Rosskopf (Ed.), Children’s mathematical concepts (pp. 111–144). Teachers College Press.

[bib78] Tall, D. (1980). The notion of infinite measuring number and its relevance in the intuition of infinity. Educational Studies in Mathematics, 11(3), 271–284. 10.1007/BF00697740

[bib44] Tillman, K. A., & Barner, D. (2015). Learning the language of time: Children’s acquisition of duration words. Cognitive Psychology, 78, 57–77. 10.1016/j.cogpsych.2015.03.001, 25867093

[bib45] Tillman, K., Fukuda, E., & Barner, D. (2022). Children gradually construct spatial representations of temporal events. Child Development, 93(5), 1380–1397. 10.1111/cdev.13780, 35560030

[bib80] Tillman, K., Tulagan, N., Fukuda, E., & Barner, D. (2018). The mental timeline is gradually constructed in childhood. Developmental Science, 21(6), Article e12679. 10.1111/desc.12679, 29749676

[bib46] Vandierendonck, A., Kemps, E., Fastame, M. C., & Szmalec, A. (2004). Working memory components of the Corsi blocks task. British Journal of Psychology, 95(Pt 1), 57–79. 10.1348/000712604322779460, 15005868

[bib47] Wagner, K., Tillman, K., & Barner, D. (2016). Inferring number, time, and color concepts from core knowledge and linguistic structure. In D. Barner & A. S. Baron (Eds.), Core knowledge and conceptual change (pp. 105–126). Oxford University Press. 10.1093/acprof:oso/9780190467630.003.0007

[bib81] Walsh, V. (2003). A theory of magnitude: Common cortical metrics of time, space, and quantity. Trends in Cognitive Sciences, 7(11), 483–488. 10.1016/j.tics.2003.09.002, 14585444

[bib48] Winter, B., Marghetis, T., & Matlock, T. (2015). Of magnitudes and metaphors: Explaining cognitive interactions between space, time, and number. Cortex, 64, 209–224. 10.1016/j.cortex.2014.10.015, 25437376

[bib49] Wistedt, I., & Martinsson, M. (1996). Orchestrating a mathematical theme: Eleven-year olds discuss the problem of infinity. Learning and Instruction, 6(2), 173–185. 10.1016/0959-4752(96)00001-1

[bib82] Wynn, K. (1990). Children’s understanding of counting. Cognition, 36(2), 155–193. 10.1016/0010-0277(90)90003-3, 2225756

[bib83] Wynn, K. (1992). Child’s acquisition of the number words and the counting system. Cognitive Psychology, 24(2), 220–251. 10.1016/0010-0285(92)90008-P

