# Supplementary material for: Everything is Infinite: Children’s Beliefs About Endless Space, Time, and Number
Source: Open Mind (Camb). 2023 Sep 20;7:715–31. doi: 10.1162/opmi_a_00104 (PMC10575555; doi:10.1162/opmi_a_00104)
Supplement: Supplementary file 1 [file opmi-07-715-s001.docx]

[**Order Effects. 2**](#_f7uy0dqos3py)

[**Expanded Age Effects 2**](#_ykxi555mrp8b)

[Table S1. Results of logistic regression predicting binary infinity knowledge from age in months. 2](#_cwdwrong3lzf)

[Table S2. Mean age of children falling into each category of infinity knowledge. 2](#_1ojop4zj6zd)

[**Expanded reporting of frequencies of belief-level classification 3**](#_micpsocnel05)

[Table S3. Number of observations per cell 3](#_yk9gs1jh03jv)

[**Unplanned, age restricted analyses. 3**](#_gdyqgs3758th)

[**Full model reporting 4**](#_bzbmiw4z1nzg)

[Number: Add One 4](#_7znniva4qyqd)

[Number: End/Forever 4](#_q31rb2ebuvdf)

[Space: Adding 4](#_7wpz2r4hrwok)

[Space: End/Forever 4](#_7lbi8uswcri2)

[Time: Adding 5](#_5zdat8x0l2kb)

[Time: End/Forever 5](#_vr1yj7gvp50b)

[**Use of time/space/time words during infinity interview. 5**](#_i135ti9bv51u)

[Figure S1. 7](#_6xy22v90cs94)

[Figure S2. 8](#_4o4qerqcl000)

[Figure S3. 8](#_uy1m77civkk1)

[Table S4. 8](#_xou6xjqq5ajv)

[**Infinity Classification for low-scoring HSI performers 9**](#_rau377g82ngi)

[Table S5. 9](#_qd1yyra29deg)

#

# Order Effects.

We found no systematic order effects in our data, meaning that the likelihood of expressing beliefs in infinity in a particular domain did not depend on the order in which the questions were asked. Infinity classification did not differ in any domain (all *p* >.10) depending on which of 12 versions of the task the participant received.

We asked whether infinity classification differed depending on which domain participants saw first. There were no differences in level of infinity belief for space (Chi-Sq(6, 109) = 7.49, *p* = .28) or time (Chi-Sq(6, 108) = 9.53, *p* = .15) depending on which domain they were asked about first. There was a n effect of classification for the domain of number (Chi-Sq(6, 109) = 14.18, *p* = .03), such that there were more non-believers (*n* = 6) for number when space was the first domain tested than when number (*n* = 1) or time (*n* = 3) were*.* However, given the small sample sizes involved and the lack of an obvious systematic pattern for these potential order effects, we believe it is unlikely that these order effects reflect true differences due to order.

#

#

# Expanded Age Effects

For each DV (adding, endless), and for each domain (space, time, and number), we conducted logistic regressions predicting level of belief from age in months. There we no effects.

## Table S1. Results of logistic regression predicting binary infinity knowledge from age in months.

|  | Adding | Endless |
| --- | --- | --- |
| Number | *B* = .03, *SE* = .02, *p* = .11 | *B* = -.05, *SE* = .02, *p* = .09 |
| Space | *B* = .02, *SE* = .02, *p* = .29 | *B* = .02, *SE* = .02, *p* = .15 |
| Time | *B* = -.0007, *SE* = .02, *p* = .97 | *B* = -.0001, *SE* = .02, *p* = .99 |

## Table S2. Mean age of children falling into each category of infinity knowledge.

|  | Full Infinity | Endless Only | Adding Only | Non-Believer |
| --- | --- | --- | --- | --- |
| Number | 73.5 m | 64.9 m. | 68.1 m. | 67.9 m |
| Space | 69.95 m. | 68.4 m. | 74.3 m. | 67.4 m. |
| Time | 70.27 m . | 70.42 m. | 69.6 m. | 70.10 m. |

# Expanded reporting of frequencies of belief-level classification

## Table S3. Number of observations per cell

|  | Number | | Space | | Time | |
| --- | --- | --- | --- | --- | --- | --- |
|  | Goes on Forever | Ends | Goes on Forever | Ends | Goes on Forever | Ends |
| Can Add 1 | *N* = 54 | *N* = 34 | *N* = 60 | *N* = 26 | *N* = 56 | *N* = 30 |
| Can’t Add 1 | *N* = 11 | *N* = 10 | *N* = 18 | *N* = 5 | *N* = 12 | *N* = 10 |

# Unplanned, age restricted analyses.

Previous studies in slightly younger children have found that measures of counting ability, and sometimes age, are related to infinity knowledge in the case of number. Because these studies differed both with respect to the ages of children and the measures they used, we asked whether effects might emerge in our dataset if we restricted analyses to younger age groups. To do so, we conducted post hoc analyses focusing only on our 4- to 6-year-old children (*N* = 80). Once again, we found that English Next Number performance was a significant predictor of the belief that it was always possible to add 1 to the biggest number (Chi-Sq = 5.56, *p* = .018), and that there were no other predictors of this infinity belief. As with our planned analyses, we found no other effects of our predictors on infinity beliefs except that there was an effect of age on children’s belief that space was endless (Chi-Sq = 4.54, *p* = .03), though in an unexpected direction (older children were more likely than younger children to report that space ends).

# Full model reporting

## Number: Add One

| **Term** | **Estimate** | **Std Error** | **ChiSquare** | **Prob>ChiSq** |
| --- | --- | --- | --- | --- |
| **Intercept** | **-2.6873733** | **2.4337342** | **1.22** | **0.2695** |
| **Corsi** | **-0.0603077** | **0.1609532** | **0.14** | **0.7079** |
| **Initial Highest Count (highest number reached without error)** | **0.00218434** | **0.0120947** | **0.03** | **0.8567** |
| **NNScore** | **-0.481565** | **0.1777057** | **7.34** | **0.0067*** |
| **Final Highest Count (highest number reached)** | **0.026005** | **0.0161675** | **2.59** | **0.1077** |
| **Age in months** | **0.09348999** | **0.0457347** | **4.18** | **0.0409*** |

## Number: End/Forever

| **Term** | **Estimate** | **Std Error** | **ChiSquare** | **Prob>ChiSq** |
| --- | --- | --- | --- | --- |
| **Intercept** | **-2.044501** | **1.8165924** | **1.27** | **0.2604** |
| **Corsi** | **-0.1325189** | **0.1168548** | **1.29** | **0.2568** |
| **Initial Highest Count (highest number reached without error)** | **-0.0092618** | **0.0088063** | **1.11** | **0.2929** |
| **NNScore** | **-0.0552442** | **0.1150928** | **0.23** | **0.6312** |
| **Final Highest Count (highest number reached)** | **-0.0022581** | **0.0107691** | **0.04** | **0.8339** |
| **Age in months** | **0.05200858** | **0.0337858** | **2.37** | **0.1237** |

## Space: Adding

| **Term** | **Estimate** | **Std Error** | **ChiSquare** | **Prob>ChiSq** |
| --- | --- | --- | --- | --- |
| **Intercept** | **0.16209808** | **2.2417102** | **0.01** | **0.9424** |
| **Corsi** | **-0.0103958** | **0.1433739** | **0.01** | **0.9422** |
| **Initial Highest Count (highest number reached without error)** | **-0.0030698** | **0.0114834** | **0.07** | **0.7892** |
| **NNScore** | **0.00624653** | **0.1467921** | **0.00** | **0.9661** |
| **Final Highest Count (highest number reached)** | **0.00259204** | **0.0138221** | **0.04** | **0.8512** |
| **Age in months** | **0.01976507** | **0.040675** | **0.24** | **0.6270** |

## Space: End/Forever

| **Term** | **Estimate** | **Std Error** | **ChiSquare** | **Prob>ChiSq** |
| --- | --- | --- | --- | --- |
| **Intercept** | **-3.2403426** | **1.8250677** | **3.15** | **0.0758** |
| **Corsi** | **-0.1092566** | **0.1163365** | **0.88** | **0.3477** |
| **Initial Highest Count (highest number reached without error)** | **-0.0042672** | **0.0087026** | **0.24** | **0.6239** |
| **NNScore** | **-0.1108116** | **0.1204998** | **0.85** | **0.3578** |
| **Final Highest Count (highest number reached)** | **0.00387153** | **0.0109733** | **0.12** | **0.7242** |
| **Age in months** | **0.05900744** | **0.0336766** | **3.07** | **0.0797** |

## Time: Adding

| **Term** | **Estimate** | **Std Error** | **ChiSquare** | **Prob>ChiSq** |
| --- | --- | --- | --- | --- |
| **Intercept** | **4.31965556** | **2.4002676** | **3.24** | **0.0719** |
| **Corsi** | **0.0720926** | **0.1382825** | **0.27** | **0.6021** |
| **Initial Highest Count (highest number reached without error)** | **0.00864141** | **0.0113822** | **0.58** | **0.4477** |
| **NNScore** | **0.1888383** | **0.1424821** | **1.76** | **0.1851** |
| **Final Highest Count (highest number reached)** | **-0.0039258** | **0.0133764** | **0.09** | **0.7692** |
| **Age in months** | **-0.0720498** | **0.0436461** | **2.73** | **0.0988** |

## Time: End/Forever

| **Term** | **Estimate** | **Std Error** | **ChiSquare** | **Prob>ChiSq** |
| --- | --- | --- | --- | --- |
| **Intercept** | **-2.0474644** | **1.7448253** | **1.38** | **0.2406** |
| **Corsi** | **-0.1362323** | **0.1191046** | **1.31** | **0.2527** |
| **Initial Highest Count (highest number reached without error)** | **-0.0103112** | **0.0085995** | **1.44** | **0.2305** |
| **NNScore** | **0.02120413** | **0.1142337** | **0.03** | **0.8527** |
| **Final Highest Count (highest number reached)** | **0.00536753** | **0.0106004** | **0.26** | **0.6126** |
| **Age in months** | **0.03452614** | **0.0316646** | **1.19** | **0.2756** |

# Use of time/space/time words during infinity interview.

As preregistered, we asked whether children disproportionately referenced a particular domain when they were asked to generate the largest X they could think of during the infinity interview. Because children could choose from the choices provided by the experimenter, we would predict, *a priori*, very high levels of within-domain responses, and that the levels of across-domain responses (e.g,. referencing number when asked about space) would be quite low, since none of the choices/prompts provided by the experimenter included cross-domain reference..

In general, we found that children typically referenced the domain under consideration explicitly, and that when they also referenced other domains, they typically did so by referencing number (see below). In general, these data were challenging to code -- this is in part because children’s spontaneous speech can be challenging to code, in part because of the ambiguity of *a* vs. *one* (items like “a million years” were coded as containing both time and number, while “a year” was coded as only containing time), and in part because of the high prevalence of words and images that can refer to multiple domains (e.g., “The whole universe til the end of the world”; “half past 30”)

**
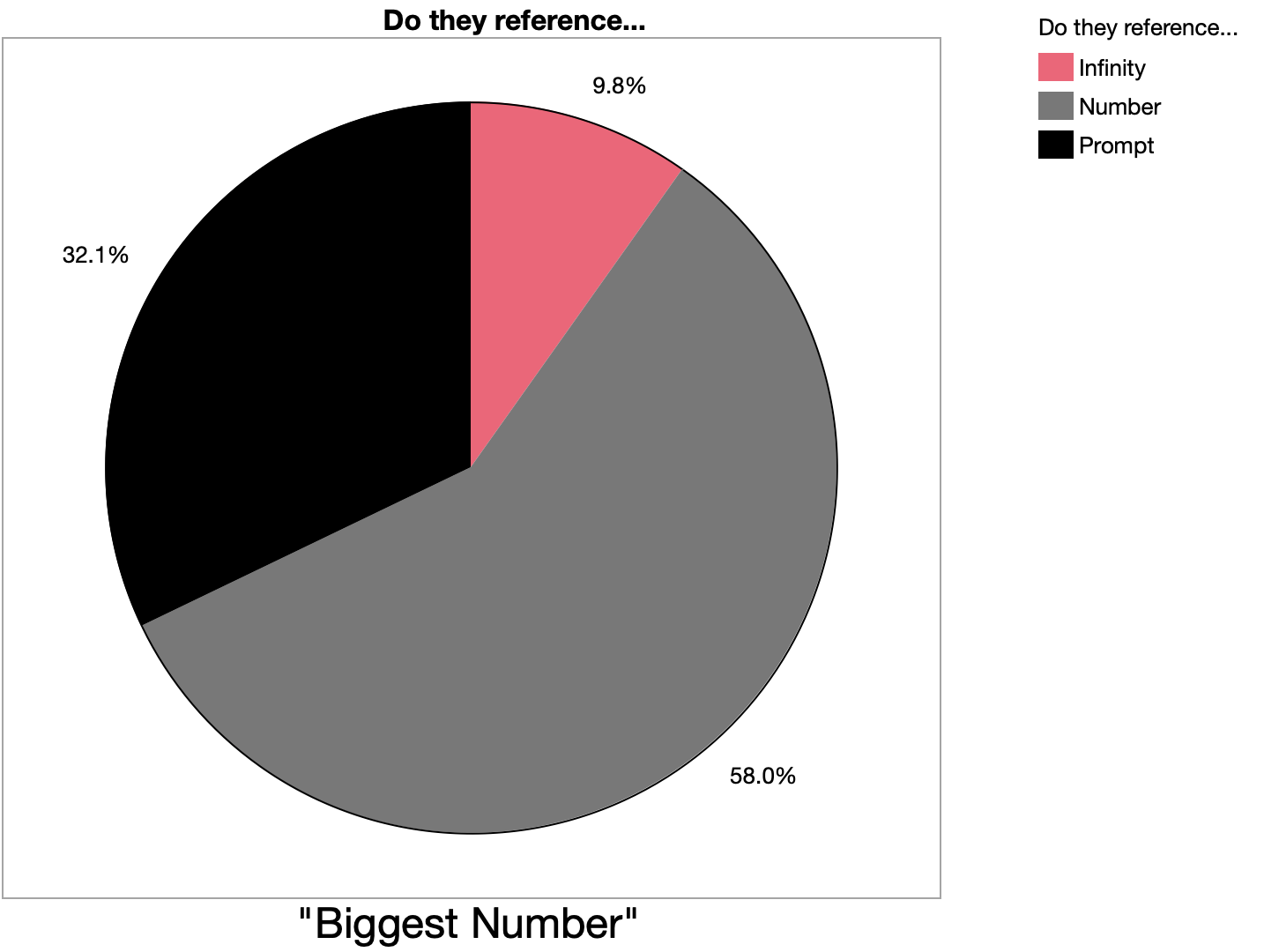
**

## Figure S1.

**
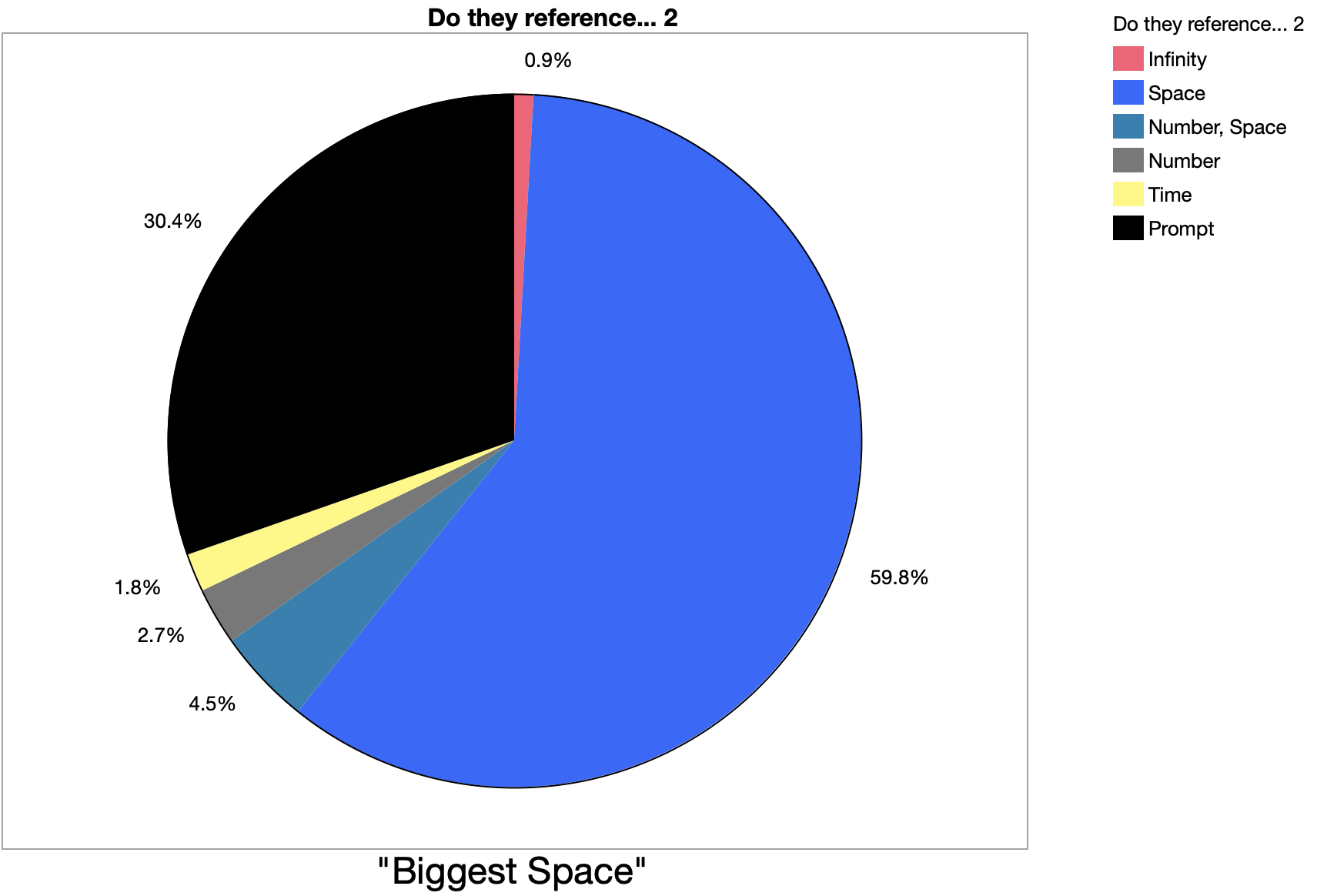
**

## Figure S2.

**
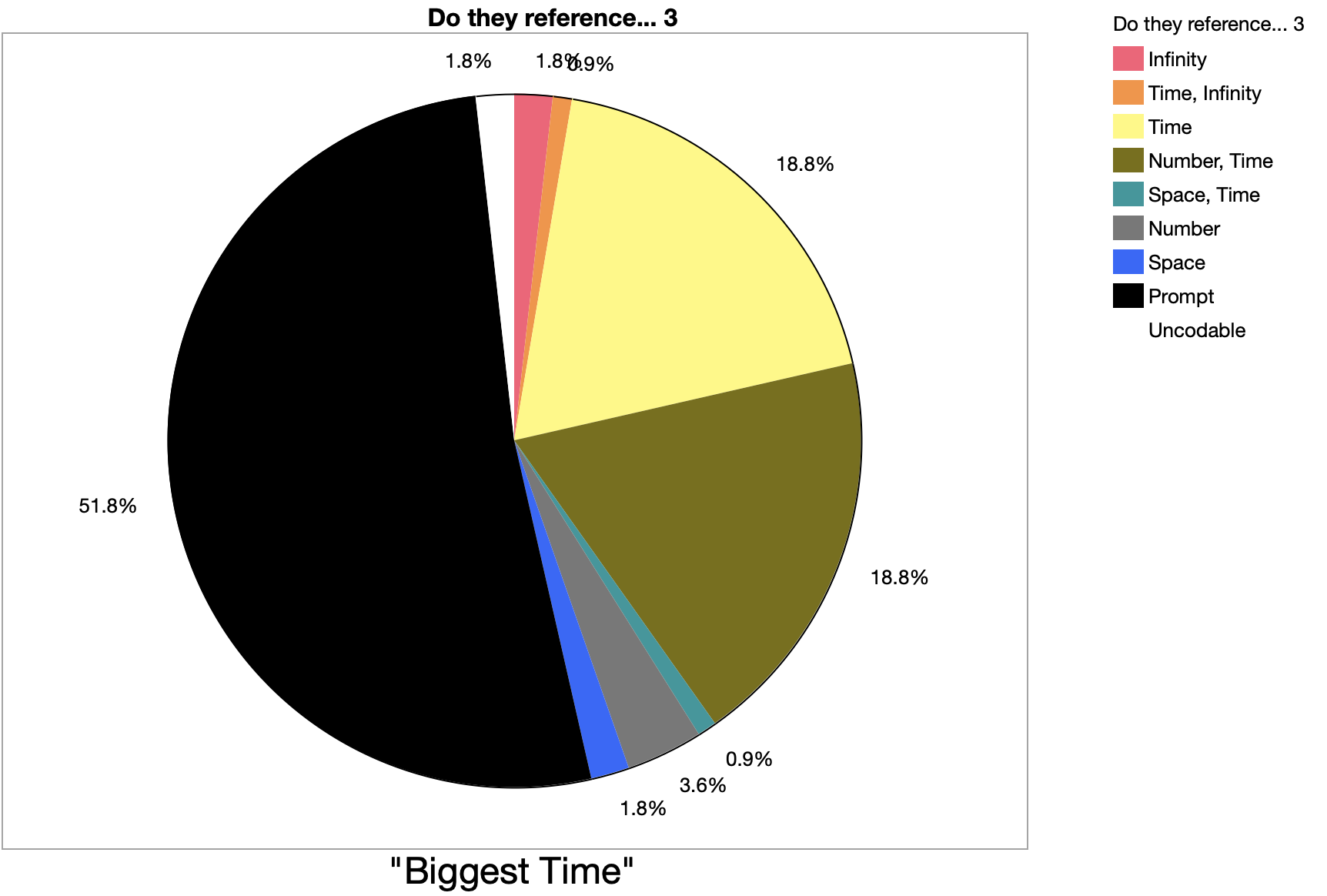
**

## Figure S3.

We wish to note that it was strikingly challenging to code these data, and we suspect that reasonable individuals might disagree about the coding described in figures S3-S5 above. Indeed, as is noted in the main text, it is often challenging to identify which domain(s) is/are being referenced in a given utterance.

## Table S4.

| **Response to “what’s the biggest time you can think about?” and [annotations]** |
| --- |
| “a month” [just time, but if they had said “one month” it would have been number, too!] |
| “a zillion days” [number and time, even though ‘zillion’ isn’t a real number] |
| “a century” [is a century just a time word, or does its precise numerical meaning also make it a number word? ] |
| “a roadtrip to Texas” [total space/time conflation] |
| “Going to the beach on my birthday” [too unclear] |
| “1,000 times” [are they using time as a unit of time measure? something else?] |
| “if the world came in 1000 days” [definitely time and number, maybe also space?] |

# Infinity Classification for low-scoring HSI performers

In response to reviewer comments, we conducted post-hoc analyses of the 10 children who were included in our final dataset, but who did not provide adult-like responses on at least ¾ items in the HSI interview.

## Table S5.

|  | Number Classification | Space Classification | Time Classification |
| --- | --- | --- | --- |
| Not enough data on the HSI task (*N* = 1) | Full Infinity: 1 | Full Infinity: 1 | Full Infinity: 1 |
| Answered 2 or fewer HSI questions correctly (*N* = 9) | Non-Believer: 1  Endless Only: 2  Adding Only: 3  Full Infinity: 3 | Non-Believer: 1  Endless Only: 0  Adding Only: 0  Full Infinity: 7 | Non-Believer: 2  Endless Only: 1  Adding Only: 0  Full Infinity: 6 |
